# PuWRKY31 affects ethylene production in response to sucrose signal in pear fruit

**DOI:** 10.1093/hr/uhac156

**Published:** 2022-08-26

**Authors:** Xinyue Li, Wei Guo, Mingyang Xu, Jiaming Zhao, Guan Wang, Hui Yuan, Aide Wang

**Affiliations:** Key Laboratory of Fruit Postharvest Biology (Liaoning Province), Key Laboratory of Protected Horticulture (Ministry of Education), National & Local Joint Engineering Research Center of Northern Horticultural Facilities Design & Application Technology (Liaoning), College of Horticulture, Shenyang Agricultural University, Shenyang, 110866, China; Key Laboratory of Fruit Postharvest Biology (Liaoning Province), Key Laboratory of Protected Horticulture (Ministry of Education), National & Local Joint Engineering Research Center of Northern Horticultural Facilities Design & Application Technology (Liaoning), College of Horticulture, Shenyang Agricultural University, Shenyang, 110866, China; Key Laboratory of Fruit Postharvest Biology (Liaoning Province), Key Laboratory of Protected Horticulture (Ministry of Education), National & Local Joint Engineering Research Center of Northern Horticultural Facilities Design & Application Technology (Liaoning), College of Horticulture, Shenyang Agricultural University, Shenyang, 110866, China; Key Laboratory of Fruit Postharvest Biology (Liaoning Province), Key Laboratory of Protected Horticulture (Ministry of Education), National & Local Joint Engineering Research Center of Northern Horticultural Facilities Design & Application Technology (Liaoning), College of Horticulture, Shenyang Agricultural University, Shenyang, 110866, China; Institute of Soybean, Heilongjiang Academy of Agricultural Sciences, 368 Xuefu Road, Harbin, 150086, China; Key Laboratory of Fruit Postharvest Biology (Liaoning Province), Key Laboratory of Protected Horticulture (Ministry of Education), National & Local Joint Engineering Research Center of Northern Horticultural Facilities Design & Application Technology (Liaoning), College of Horticulture, Shenyang Agricultural University, Shenyang, 110866, China; Key Laboratory of Fruit Postharvest Biology (Liaoning Province), Key Laboratory of Protected Horticulture (Ministry of Education), National & Local Joint Engineering Research Center of Northern Horticultural Facilities Design & Application Technology (Liaoning), College of Horticulture, Shenyang Agricultural University, Shenyang, 110866, China

## Abstract

The ripening of climacteric fruits is mainly controlled by the plant hormone ethylene. The regulatory effect of sucrose on ethylene biosynthesis in fruits remains unclear. Here we examined ethylene production in two Ussurian pear (*Pyrus ussuriensis*) varieties, ‘Nanguo’ (NG) pear and its bud sport variety (BNG), which has a higher sucrose content. We found that the peak of ethylene release occurred earlier in BNG fruit than in NG fruit during ripening. The expression of the transcription factor *PuWRKY31* was higher in BNG fruit than in NG fruit, and was induced by sucrose treatment. Furthermore, PuWRKY31 bound to the promoters of ethylene biosynthetic genes and upregulated their transcription. Our findings suggest a mechanism by which sucrose regulates ethylene biosynthesis in pears.

## Introduction

Fleshy fruit ripening is a key physiological process with irreversible alterations in sugar content, texture, color, aroma, and nutritional components, and is of great significance to the human diet [1]. Fruit ripening is classified as non-climacteric or climacteric. In climacteric fruits, the ripening process is mainly controlled by ethylene and reducing the production of ethylene can significantly delay ripening and extend shelf life [2, 3].

Ethylene biosynthesis has been studied extensively [4, 5]. The formation of 1-aminocyclopropane-1-carboxylic acid (ACC) by the enzyme ACC synthase (ACS; EC 4.1.1.14) from *S*-adenosyl methionine initiates the biosynthesis of ethylene. Then, ACC oxidase (ACO) oxidizes ACC to form ethylene. These two steps are the key processes of the Yang cycle [6]. The important roles of *ACS* and *ACO* in fruit ripening have been well studied. In tomato (*Solanum lycopersicum*), mutants with higher SlACS2 protein levels showed overproduction of ethylene and accelerated fruit ripening, whereas mutants with lower SlACS2 protein levels showed prolonged fruit ripening [7]. In transgenic apples, silencing of *MdACS1* or *MdACO1* greatly reduced ethylene production [8, 9].

Ethylene is modulated by various factors, including internal cues such as development, plant hormones, and plant nutrition as well as environmental factors, such as light and temperature [2]. Sucrose plays a role in many developmental processes in plants, including fruit ripening. Sucrose treatment inhibits activity of ACS and ACO, leading to a decline in ethylene productionin climacteric-like cut carnations (*Dianthus caryophyllus* cv. ‘Barbara’) [10]. In tomato fruit, exogenous sucrosetreatment accelerates the ripening process by enhancing ethylene biosynthesis and upregulating the expression of ethylene biosynthesis-related genes [11]. Sucrose can significantly promote the ripening of strawberry(*Fragaria ananassa*) [12], which is a non-climacteric fruit.However, the molecular mechanism through which sucrose regulates ethylene biosynthesis remains unclear.

The WRKY transcription factors are plant-specific, contain a highly conserved WRKY domain that binds to the target gene, W-box, and play a central role in variousprocesses of plant growth, development, and resistance. In our previous study, we used ‘Nanguo’ pear (NG), which is a local variety of Ussurian pear (*Pyrus ussuriensis*), and its bud sport variety BNG, which accumulates a higher content of sucrose, as experimental subjects. Weobserved that the higher sucrose content in BNG fruitwas due to the higher expression of *PuWRKY31*. Further evidence showed that high expression of *PuWRKY31* results from high histone acetylation levels [13]. Here,we verified the effects of sucrose signals on ethylene biosynthesis in pears (*P. ussuriensis*). The expression of *PuWRKY31* was significantly promoted in pears treated with exogenous sucrose, and PuWRKY31 upregulated ethylene biosynthetic genes to enhance ethylene biosynthesis and ripening of pear fruit.

## Results

### BNG fruit has a rapid-ripening phenotype

**Figure 1:**
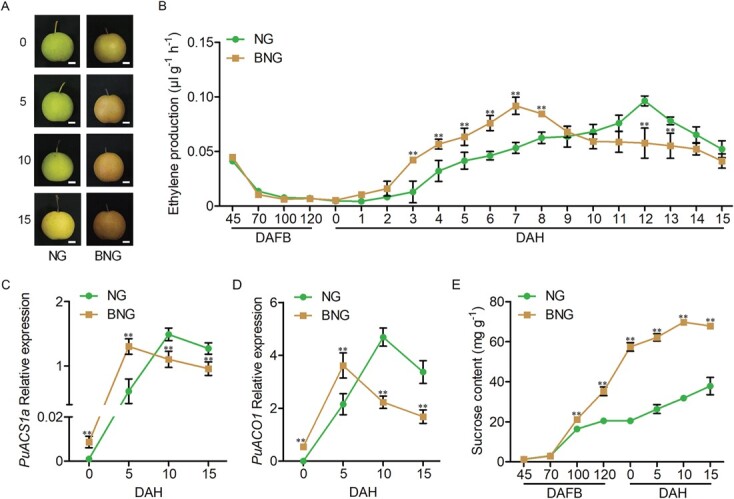
BNG fruit has a more rapid ripening phenotype than NG fruit. NG and BNG fruits were collected on different days after full bloom. Fruits collected in 2018 were stored at room temperature for 15 days (A). Ethylene production (B), expression levels of *PuACS1a* (C) and *PuACO1* (D), and sucrose content (E) were determined. All data are shown as mean ± standard error collected from three biological replicates. Asterisks indicate significant differences as determined by Student’s *t*-test (^**^P < .01).

In this research, we investigated the ethylene production of NG and BNG fruits during fruit ripening. After harvest, the fruits were stored at 25°C for 15 days, during which the ripening process of the fruits was finished. During this period, the firmness and ethylene production of the fruits were measured to estimate fruit ripening. We found that the firmness of both NG and BNG fruits was ~80 N at harvest (day 0, Supplementary Data Fig. S1A and B), and it gradually decreased during storage, while the firmness of the BNG fruit dropped more rapidly (Supplementary Data Fig. S1A and B). Regarding ethylene production, there was no significant difference between NG and BNG fruits during development, while both varieties showed a peak of ethylene emission during ripening, and the peak appeared much earlier in BNG fruit than in NG fruit (Fig. 1A and B; Supplementary Data Fig. S1C). These results indicate that BNG fruit has a more rapid ripening phenotype. *PuACS1a* and *PuACO1* were shown to be indispensable for ethylene biosynthesis in pears by Ji *et al*. [14]. We examined the expression of *PuACS1a* and *PuACO1* by reverse transcription–quantitative PCR (RT–qPCR). We found that the expression level of *PuACS1a* and *PuACO1* was dramatically higher in BNG fruit than in NG fruit from 0 to 5 days after harvest (DAH), but lower from 10 to 15 DAH (Fig. 1C and D; Supplementary Data Fig. S1D and E). These results are in agreement with those related to the changes in ethylene production (Fig. 1B; Supplementary Data Fig. S1C).

**Figure 2:**
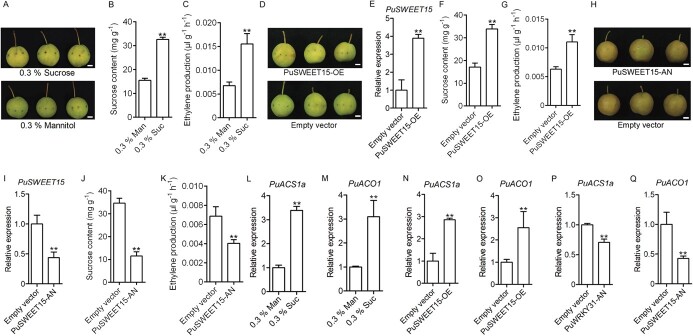
Sucrose promotes the production of ethylene. (A–C) On-tree NG fruits were treated with 0.3% sucrose by injecting sucrose into fruits at 120 DAFB and injected fruits were harvested 6 days after injection. Fruits injected with 0.3% mannitol were used as control (A). After treatment, the sucrose content (B) and ethylene production were measured (C). (D–G) *PuSWEET15* was overexpressed in on-tree NG fruits at 120 DAFB, the infiltrated fruit were harvested 6 days after infiltration, and the empty pRI101 vector was used as a control (D). After treatment, the expression level of *PuSWEET15* (E), sucrose content (F), and ethylene production (G) were measured. H-K. *PuSWEET15* was silenced in on-tree BNG fruits at 120 DAFB, the infiltrated fruits were harvested 6 days after infiltration, and the empty pRI101 vector was used as a control (H). After treatment, the expression level of *PuSWEET15* (I), sucrose content (J), and ethylene production (K) were measured. (L, M) Expression levels of *PuACS1a* (L) and *PuACO1* (M) in fruits treated with 0.3% sucrose and 0.3% mannitol. (N, O) Expression levels of *PuACS1a* (N) and *PuACO1* (O) in *PuSWEET15*-overexpressing and control fruits. (P, Q) Expression levels of *PuACS1a* (P) and *PuACO1* (Q) in *PuSWEET15*-silenced and control fruits. All data are mean ± standard error from three biological replicates. Asterisks indicate significant differences as determined by Student’s *t*-test (^**^P < .01).

Our previous study showed that BNG had higher sucrose accumulation during development [13]. In this study, we compared the sucrose content of the NG and BNG fruits. We observed a dramatically higher sucrose content in BNG fruit than in NG fruit from 100 to 120 days after full bloom (DAFB) and during ripening (Fig. 1E). We hypothesized that sucrose accumulation played a vital role in regulating the ripening process of BNG fruit.

### Sucrose promotes the production of ethylene

To elucidate whether sucrose regulates pear fruit ripening, we performed a pharmacological experiment, in which exogenous sucrose (CAS:57-50-1) was introduced by injection into on-tree NG fruits at 120 DAFB. Fruits injected with mannitol (CAS:69–65-8) were used as a control. As shown in Fig. 2A, the fruits started to ripen (turn yellow) 5 days after sucrose supplementation, while the fruits treated with mannitol were still green. Sucrose treatment significantly enhanced sucrose content and ethylene content (Fig. 2B and C). These results clearly indicate that sucrose plays a crucial role in regulating pear fruit ripening.

We previously proved that *PuSWEET15* plays a vital role in higher sucrose accumulation in pear fruits [13]. So to understand the relationship between sucrose and ethylene, we generated a *PuSWEET15* overexpression vector and overexpressed it in NG fruit by *Agrobacterium tumefaciens*-mediated infiltration. *PuSWEET15*-overexpressing (PuSWEET15-OE) fruits started to turn yellow 5 days after infiltration (Fig. 2D). The expression of *PuSWEET15* was higher in PuSWEET15-OE fruits (Fig. 2E). The sucrose content in PuSWEET15-OE fruits was observably higher than in control fruits (Fig. 2F), and ethylene production was greatly increased in PuSWEET15-OE fruits (Fig. 2G). Silencing of *PuSWEET15* (PuSWEET15-AN) in BNG fruits (Fig. 2H and I) resulted in considerably reduced sucrose and ethylene contents in PuSWEET15-AN fruits (Fig. 2J and K). These results corroborated the finding that sucrose contributes to ethylene biosynthesis.

We then investigated the influence of sucrose on the expression of the key genes in the ethylene synthesis pathway. The results indicated that the expression of both *PuACS1a* and *PuACO1* was greatly enhanced by exogenous sucrose supplementation (Fig. 2L and M), and these genes were highly expressed in PuSWEET15-OE fruits (Fig. 2N and O). The expression of these genes was considerably reduced in PuSWEET15-AN fruits (Fig. 2P and Q). Taken together, these results indicated that sucrose functions as a signal and thereby plays a pivotal role in regulating the expression of *PuACS1a* and *PuACO1* to promote the biosynthesis of ethylene during pear fruit ripening.

### *PuWRKY31* is highly expressed in BNG fruits and regulates the expression of ethylene biosynthetic genes

To illustrate the expression profiles of *PuACS1a* and *PuACO1*, we cloned the CDS (coding sequence) of these two genes and found no difference in the CDS of these two genes (Supplementary Data Figs S2 and S3). Moreover, the promoter regions of *PuACS1a* and *PuACO1* in NG and BNG fruits were the same as each other (Supplementary Data Figs S4 and S5).

**Figure 3:**
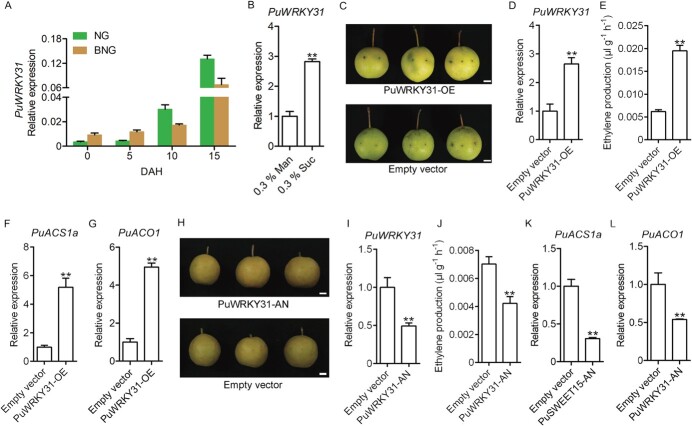
Functional analysis of transcription factor *PuWRKY31*. (A) Expression level of *PuWRKY31* in NG and BNG fruits during ripening in 2018. (B) Expression level of *PuWRKY31* in sucrose-treated NG fruits. (C–G) *PuWRKY31* was overexpressed in on-tree NG fruits at 120 DAFB. The infiltrated fruits were harvested 6 days after infiltration, and the empty pRI101 vector was used as a control (C). After treatment, the expression level of *PuWRKY31* (D), ethylene production (E), and expression levels of *PuACS1a* (F) and *PuACO1* (G) were investigated in *PuWRKY31*-overexpressing and control fruits. (H–L) *PuWRKY31* was silenced in on-tree BNG fruits at 120 DAFB, the infiltrated fruit were harvested 6 days after infiltration, and the empty pRI101 vector was used as a control (H). After treatment, the expression level of *PuWRKY31* (I), ethylene production (J), and expression levels of *PuACS1a* (K) and *PuACO1* (L) were measured. PuWRKY31-OE, *PuWRKY315* overexpressing fruit; Empty vector, control fruit overexpressing empty pRI101 vector; PuWRKY31-AN, *PuWRKY31* silenced fruit. All data are mean ± standard error from three biological replicates. Asterisks indicate significant differences as determined using Student’s *t*-test (^**^P < .01).

Next, we found that *PuACS1a* and *PuACO1* promoters have some binding sites (*cis*-elements) for transcription factors, such as WRKY, DOF (DNA-binding one finger), and MYB. Our previous study showed that, compared with NG fruits, *PuWRKY31* showed higher expression levels in BNG fruits during development [13]. Here we observed that *PuWRKY31* was also expressed at higher levels in BNG fruits during the early storage period (Fig. 3A; Supplementary Data Fig. S6). Further, we found that the expression of *PuWRKY31* was observably induced by exogenous sucrose supplementation (Fig. 3B). These results implied that *PuWRKY31* might function as a regulator in the sucrose signaling pathway and participate in regulating pear fruit ripening.

To further clarify the potential role of *PuWRKY31* in promoting ethylene production, we overexpressed *PuWRKY31* in NG fruits (PuWRKY31-OE) and found that the PuWRKY31-OE fruits started to turn yellow, whereas the control fruits overexpressing FLAG alone were still green (Fig. 3C). *PuWRKY31* showed a dramatically higher expression level in PuWRKY31-OE fruits than in control fruits (Fig. 3D). The high expression of *PuWRKY31* promoted the production of ethylene and *PuACS1a* and *PuACO1* expression levels (Fig. 3E–G). Silencing of *PuWRKY31* in BNG fruits (Fig. 3H and I) considerably reduced the production of ethylene and the expression levels of *PuACS1a* and *PuACO1* (Fig. 3J–L). These results implied that PuWRKY31 plays a vital role in regulating the transcription of ethylene biosynthetic genes.

### PuWRKY31 upregulates *PuACS1a* and *PuACO1* transcription by binding to their promoters

**Figure 4:**
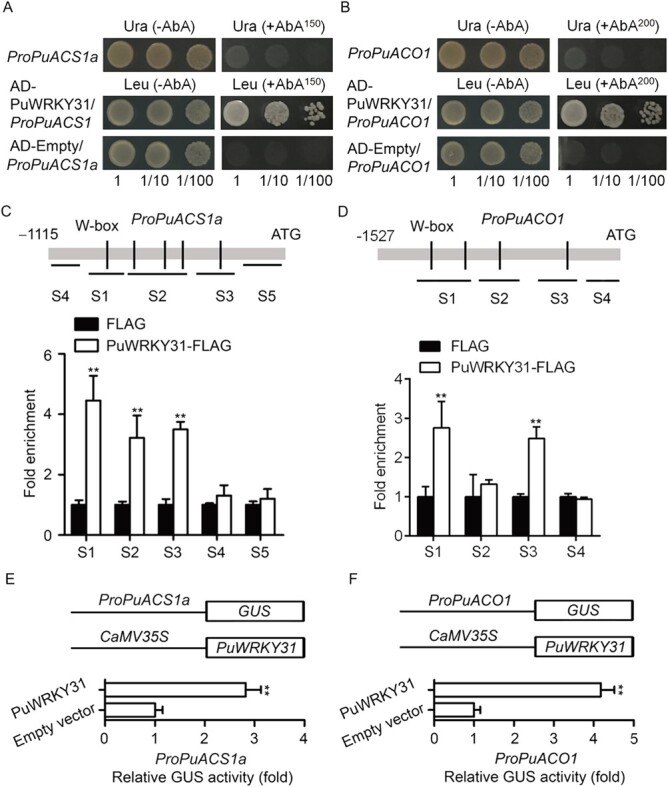
PuWRKY31 promotes the transcription of *PuACS1a* and *PuACO1*. (A, B) Y1H analysis of PuWRKY31 binding to the promoters of *PuACS1a* (*ProPuACS1a*) (A) and *PuACO1* (*ProPuACO1*). A Y1H assay was performed over a range of yeast concentrations. (C, D) ChIP–PCR showed PuWRKY31 could bind to the promoters of *PuACS1a* (*ProPuACS1a*) (C) and *PuACO1* (*ProPuACO1*) (D) *in vivo*. Values are the percentage of DNA fragments that co-immunoprecipitated with FLAG antibody relative to input DNA in PuWRKY31-OE or negative control fruits. (E, F) GUS activity analysis showed that PuWRKY31 promotes the transcription of *PuACS1a* and *PuACO1*. All data are mean ± standard error from three biological replicates. Asterisks indicate significant differences as determined by Student’s *t*-test (^**^P < .01).

The *cis*-element motifs existing in the *PuACS1a* and *PuACO1* promoters were predicted through the online database PlantCARE (http://bioinformatics.psb.ugent.be/webtools/plantcare/html/). The binding of PuWRKY31 to the *PuACS1a* and *PuACO1* promoters was verified by the yeast one-hybrid (Y1H) assay and it was found that PuWRKY31 could bind to their promoters *in vitro* (Fig. 4A and B). Chromatin immunoprecipitation–PCR (ChIP–PCR) was performed to study the binding of *PuACS1a* and *PuACO1* promoters by PuWRKY31 *in vivo*. The cross-linked chromatin samples were extracted from *PuWRKY31-FLAG*-overexpressing NG fruits (Fig. 3C). The chromatin was sonicated into 200- to 500-bp fragments and was then immunoprecipitated using an anti-FLAG antibody. Then, eluted DNA was used to amplify sequences neighboring the W-box by qPCR. Fruits overexpressing the FLAG sequence were used as negative control. We found that the S1, S2, and S3 fragments of *PuACS1a* promoters were enriched in the presence of PuWRKY31 (Fig. 4C), and *PuACO1* promoter S1 and S3 fragments were enriched in the presence of PuWRKY31 (Fig. 4D). These results indicated that PuWRKY31 also could bind to the *PuACS1a* and *PuACO1* promoters *in vivo*.

Regulation of the *PuACS1a* and *PuACO1* promoters by PuWRKY31 was then investigated by using a β-glucuronidase (GUS) activation assay, showing that PuWRKY31 induced the expression of *PuACS1a* and *PuACO1* (Fig. 4E and F). Collectively, our results suggest that PuWRKY31 binds to the promoters of *PuACS1a* and *PuACO1* and promotes their transcription*.*

## Discussion

Ethylene is indispensable for the ripening process of climacteric fruits. Sucrose is an important factor affecting fruit quality. However, whether sucrose plays a pivotal role in the biosynthesis of ethylene remains unclear. We previously reported that the BNG pear is a bud sport variety of NG pear. BNG fruits accumulate a higher level of sucrose during development than NG fruits [13].

Here, we found that BNG fruits ripened more rapidly than NG fruits (Fig. 1). To explore whether sucrose is involved in regulating ethylene production, we identified that the transcription factor PuWRKY31 is involved in sucrose-activated ethylene biosynthesis. The expression of *PuWRKY31* was higher in the BNG fruit after sucrose treatment. Further experiments revealed that PuWRKY31 directly bound to the promoters of both*PuACS1a* and *PuACO1* and enhanced their transcription, leading to the promotion of ethylene biosynthesis. Ourresults provide detailed information about the effect ofsucrose on ethylene biosynthesis in pear fruits.

Sugar signaling plays an important role in certain processes of plant growth and development, including fruit ripening [12, 15–17]. Regarding the regulation of ethylene biosynthesis by sugars, different sugars seem to have different functions in various horticultural crops. Glucose inhibits ethylene biosynthesis by suppressing ACO during tomato fruit ripening [18]. In *Paeonia suffruticosa* flowers, treatment with glucose delayed the increase in climacteric ethylene and ethylene production [19]. Sucrose treatment delayed ethylene biosynthesis in cut carnations [10] and enhanced ethylene production in tomato fruits [10, 11]. Our study provides evidence that sucrose can enhance ethylene production via the regulation of PuWRKY31 on ethylene biosynthetic genes in pear fruit.

WRKY transcription factor are one of the largest plant-specific transcription factor families, regulating gene expression by specifically binding W-box sequences [20]. We previously found that PuWRKY31 directly binds to the *PuSWEET15* promoter and upregulates its expression, leading to sucrose accumulation [13]. In this study, we observed that exogenous sucrose treatment promoted *PuWRKY31* expression (Fig. 3B) and ethylene production. Moreover, PuWRKY31 could bind to the promoters of both *PuACS1a* and *PuACO1* and upregulated their expression (Fig. 4). These findings suggest that PuWRKY31 regulates ethylene production in response to sucrose signals, and a feedback loop is formed between PuWRKY31 and sucrose. However, whether PuWRKY31 is indispensable in sucrose-induced ethylene biosynthesis requires further investigation.

**Figure 5:**
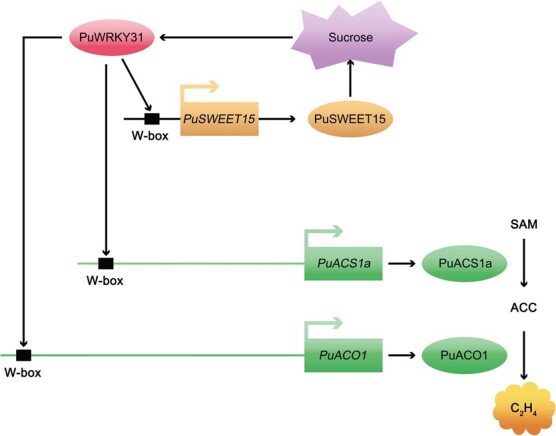
Model of PuWRKY31 inducing ethylene biosynthesis in pear fruit. Sucrose-activated PuWRKY31 binds to the promoters of *PuACS1a* and *PuACO1* to enhance their expression and increase ethylene production in pear fruit. W-box, WRKY binding site; SAM, *S*-adenosyl methionine.

Taken together, our study demonstrated how PuWRKY31 regulates pear fruit ripening (Fig. 5). Sucrose activates the expression of *PuWRKY31* and PuWRKY31 enhances the expression of *PuACS1a* and *PuACO1* by directly binding to their promoters, leading to increased ethylene production in pear fruits.

## Materials and methods

### Plant material and treatments

The pear fruits (*P. ussuriensis* NG and BNG) used in this research were supplied by the orchard of the Liaoning Pomology Institute (Xiongyue, China) at 137 and 140 DAFB (commercial harvest day) in 2018 and 2019, respectively. After harvest, the fruits were brought to the laboratory immediately and used for storage at room temperature (25°C) for 15 days and sampled every 5 days. For firmness, soluble solids and ethylene production measurement, we selected nine fruits and randomly divided them into three groups and operated as previously described [13, 21]. Firmness and soluble solid content were measured every 5 days and ethylene production was measured daily. Student’s *t*-test was used to determine statistical significance. Then, the fruits were sliced, liquid nitrogen was used to quickly freeze the fruits, and the fruits were stored at −80°C.

### Gene expression analysis, determination of fruit firmness, and ethylene production rates

Total RNA extraction, cDNA synthesis, and RT–qPCR assays were employed as described by Li *et al*. [13]. Primers used in this research are all included in Supplementary Data Set 1. The flesh firmness measurement was performed as previously described by Li *et al*. [22]. A fruit was used as one biological replicate. Three fruits were measured. For ethylene production measurement, fruits were put in a sealed container (0.86 L) equipped with a rubber stopper for 1 hour (25°C).

Then, the gas was collected by using a 1-mL syringe through the rubber stopper for the measurement of ethylene using a gas chromatograph (7890A, Agilent Technology, Santa Clara, CA, USA), as described by Li *et al*. [22]. At least six fruits were used to determine ethylene production.

### Measurements of soluble solids and sugar contents

The soluble sugar content measurement was carried out using HPLC (Agilent Technologies, 1260 Series), as previously reported by Li *et al*. [13]. First, 10 mL of 80% (v/v) ethanol was added to a centrifuge tube (50 ml), then the powder (0.3 g) was placed in the tube and the preparation was incubated in a water bath for ~30 minutes at 80°C, then centrifuged at 10 000 × g for 5 minutes to collect the supernatant. This entire process was repeated at least twice, and the supernatant collected in the above process was evaporated in boiling water until the preparation was dry. Then the sediment in the tube was dissolved in ultrapure water (1 mL) and filtered using a 0.45-μm membrane.

### Y1H assay

*PuWRKY31* was introduced into the pGADT7 vector, and the fragment of the *PuACS1a* or *PuACO1* promoter was cloned into the pAbAi vector using the restriction sites SacI and SmaI. Then we used the Matchmaker™ Gold Y1H Library Screening System kit (catalog no. 630491, Clontech) to analyze the binding of PuWRKY31 to the *PuACS1a* or *PuACO1* promoter. The culture was diluted to different fold values (×10 and ×100).

### β-Glucuronidase analysis

The reporter construct was obtained by cloning the *PuACS1a* (1115 bp) or *PuACO1* (1527 bp) promoter into the *GUS* reporter gene vector. The effector construct was generated by cloning the *PuWRKY31* CDS into the pRI101 vector. The *A. tumefaciens* strain EHA105, with the reporter or effector construct, was used to infiltrate *Nicotiana benthamiana* leaves, and GUS activity examination was performed according to the method previously described [13]. At least three independent repeated infiltrations were performed.

### *Agrobacterium*-mediated infiltration

For overexpression of *PuWRKY31* or *PuSWEET15* in NG fruit, the CDS was ligated into the plant transformation vector-pRI101 with a FLAG tag by NdeI and SacI, respectively, to form the *Pro35S:PuWRKY31-FLAG* or *Pro35S:PuSWEET15-FLAG* vector. To silence the expression of *PuWRKY31* or *PuSWEET15* in BNG fruit, a partial CDS of *PuWRKY31* or *PuSWEET15* was separately introduced into the pRI101 vector in the reverse direction to generate the antisense *PuWRKY31* construct (PuWRKY31-AN) or *PuSWEET15* construct (PuSWEET15-AN). Then, the recombinant plasmids were transformed into *A. tumefaciens* strain EHA105, and a suspension for fruit infiltration was prepared according to Li *et al*. [13]. Briefly, using the needle of a 1-mL syringe, three pinholes (depth 0.5–1 cm) were made on on-tree fruits at 120 DAFB, and then 200 μL of the infiltration buffer was infiltrated through the pinholes with a 1-mL sterile syringe. Fruits on the same branch and were selected for injection. The infiltrated fruits were harvested 6 days after infiltration and immediately carried to the laboratory. One fruit was used as a biological replicate, and at least 10 biological replicates were performed, with three fruits used for further measurement. Overexpression of *PuWRKY31* or *PuSWEET15* was performed on NG fruit and silencing of *PuWRKY31* and *PuSWEET15* on BNG fruit.

### Chromatin immunoprecipitation–PCR

The CDS of *PuWRKY31* was ligated into the pRI101-3 × FLAG vector, and then the recombinant vector was transformed into EHA105, and NG fruits were infected, as described above. ChIP assays were performed as previously described [23] with a FLAG antibody (Transgen, Beijing, China). The enrichment of immunoprecipitated chromatin was determined by qPCR. Each ChIP assay was performed three times. Five *PuACS1a* promoter regions and four *PuACO1* promoter regions were analyzed to assess their enrichment.

## Acknowledgements

This work was supported by the National Natural Science Foundation of China (32125034).

## Author contributions

A.W., H.Y., and X.L. designed this study and wrote the manuscript. X.L. and W.G. performed most of the experiments. M.X. extracted the RNA. J.Z. measured the sugar content. X.L., H.Y., W.G., M.X., and G.W analyzed the data and reviewed the article.

## Data availability

The authors confirm that all the data are available and accessible via the main text and/or the supplementary data.

## Conflict of interest

The authors declare no competing interests.

## Supplementary data

Supplementary data is available at *Horticulture Research* online.

## Supplementary Material

Web_Material_uhac156
