# Supplementary material for: PuWRKY31 affects ethylene production in response to sucrose signal in pear fruit
Source: Hortic Res. 2022 Aug 26;9:uhac156. doi: 10.1093/hr/uhac156 (PMC9533224; doi:10.1093/hr/uhac156)
Supplement: Web_Material_uhac156 [file web_material_uhac156.zip › Supplemental Figure.docx]

**Supplemental Figures**


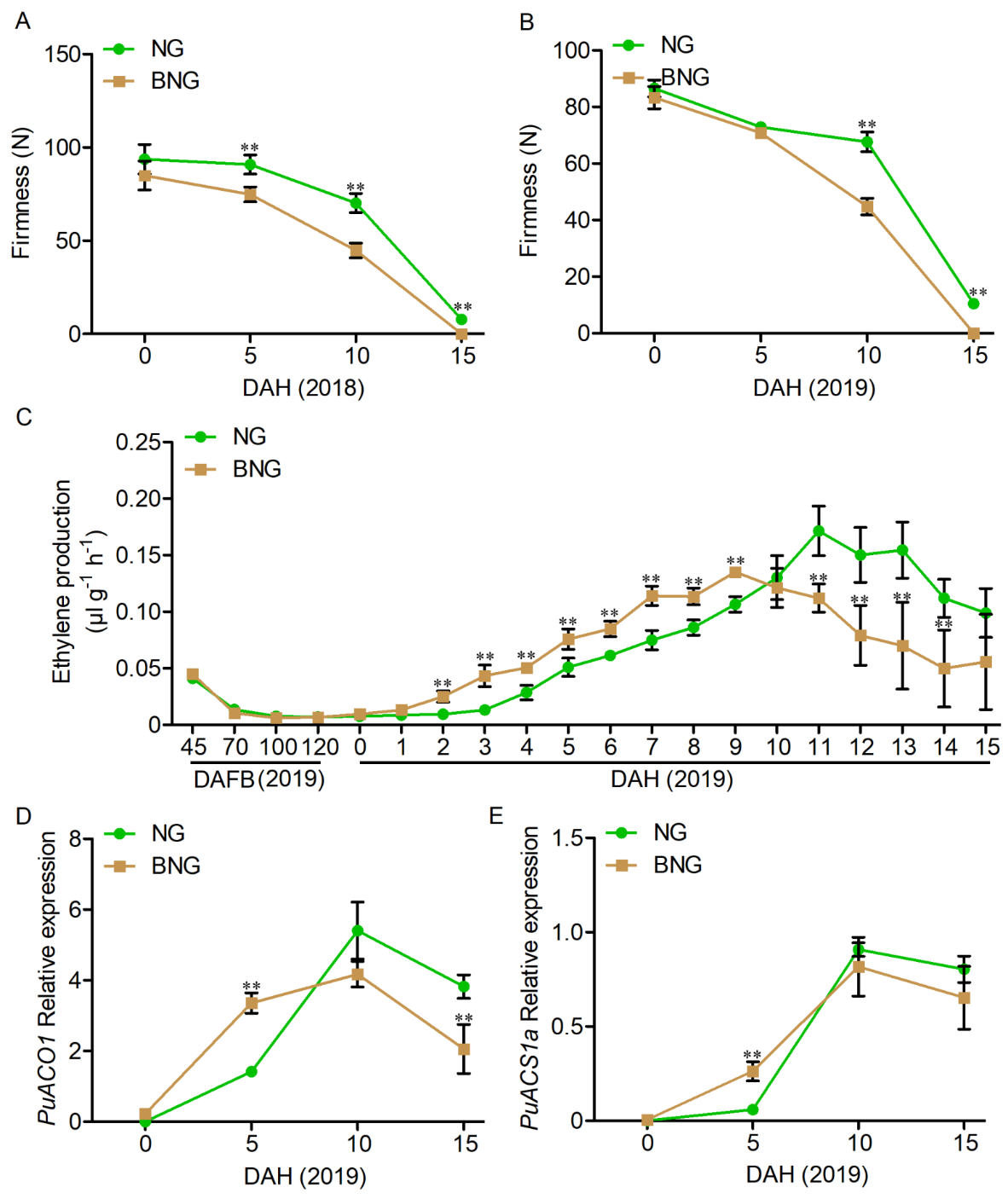


**Supplemental Figure S1.** Fruit firmness (A-B), ethylene production (C), and expression of ethylene biosynthetic gene expression (D-E) in Nanguo pear fruit (NG) and its bud sport (BNG). Fruit collected on the day of commercial harvest (137 DAFB) in 2018 and on the day of commercial harvest (135 DAFB) in 2019 were stored at room temperature for 15 days. DAFB, days after full bloom; DAH, days after harvest. All data presented are shown as mean ± SE collected from three biological replicates. Asterisks indicate significant differences as determined by Student’s *t*-test (**P < 0.01).

**Supplemental Figure S2.** Sequencing alignment results of the *PuACS1a* coding sequence in Nanguo pear fruit (NG) and its bud sport (BNG).

**Supplemental Figure S3.** Sequencing alignment results of the *PuACO1* coding sequence in Nanguo pear fruit (NG) and its bud sport (BNG).

**Supplemental Figure S4.** Sequencing alignment results of the *PuACS1a* promoter sequence in Nanguo pear fruit (NG) and its bud sport (BNG).

**Supplemental Figure S5.** Sequencing alignment results of the *PuACO1* promoter sequence in Nanguo pear fruit (NG) and its bud sport (BNG).


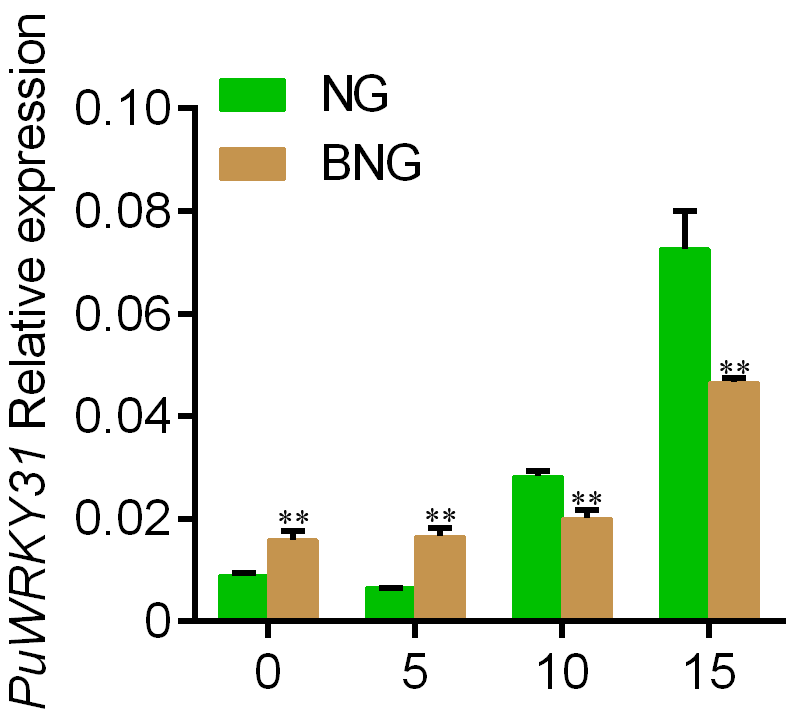


**Supplemental Figure S6.** Expression of *PuWRKY31* in Nanguo pear fruit (NG) and its bud sport (BNG) sampled in 2019 was determined by quantitative reverse transcription (qRT)-PCR.

Numbers under the x-axis indicate days after full bloom (DAH). Asterisks indicate significant differences as determined by Student’s *t-test* (**P<0.01). Three biological replicates were analyzed, and the error bars represent SE.
